# Targeting pH Inversion in Prostate Cancer Cells: A Role for Systems of Molecules of Vegetal Origin

**DOI:** 10.3390/ijms26167700

**Published:** 2025-08-08

**Authors:** Lorena Urbanelli, Krizia Sagini, Federica Delo, Sandra Buratta, Jacopo Lucci, Valentino Mercati, Carla Emiliani

**Affiliations:** 1 Department of Chemistry, Biology and Biotechnology, University of Perugia, 06100 Perugia, Perugia, Italy; krizia.sagini@medisin.uio.no (K.S.); sandra.buratta@unipg.it (S.B.); 2 Centro di Eccellenza sui Materiali Innovativi Nanostrutturati (CEMIN), University of Perugia, 06123 Perugia, Perugia, Italy; 3 Department of Pharmacology, Institute of Clinical Medicine, University of Oslo, 0372 Oslo, Norway; 4 Bios-Therapy, Physiological Systems for Health S.P.A., Loc. Aboca 20, 52037 Sansepolcro, Arezzo, Italy; fdelo@biostherapy.it (F.D.); jlucci@biostherapy.it (J.L.);

**Keywords:** pH inversion, intracellular pH, extracellular pH, lysosomal pH, herbal extracts, systems of molecules of vegetal origin

## Abstract

Intracellular alkalosis and extracellular acidosis are two pathological features associated with malignant cells. They offer advantages in terms of invasiveness and proliferation. Extracellular acidification is the consequence of intracellular metabolic changes associated with a higher metabolic rate of cancer cells, potentially inducing dangerous intracellular acidification. To overcome this menace, malignant cells adapt themselves to export hydrogen ions. Therefore, it is reasonable that targeting intracellular alkalinization and extracellular acidification to prompt the reversal of such a pH gradient towards a condition comparable to normal, untransformed cells may represent a strategy helping to contrast malignant behavior. In the present study, we investigated in vitro, in prostate cancer cell models, the biological activity towards intracellular, extracellular and organelle pH of systems of molecules of vegetal origin. A few of these systems were shown to promote intracellular acidification in vitro, whereas others were shown to prevent extracellular acidification and promote lysosomal alkalinization in a cell type-dependent manner. This result clearly indicates that these systems may function as agents interfering with malignant cells inverted pH gradient. Further analysis would be necessary to unravel the cell type specificity of their effects, as well as their mechanism of action. Nevertheless, our proof-of-principle study provides evidence that such systems of molecules can be considered interesting agents in co-adjuvating anti-cancer therapies.

## 1. Introduction

Otto Warburg first provided evidence in the 1930s that tumors, in contrast with normal tissue, have a great capacity to produce lactic acid via glycolysis, even in the presence of normal oxygen levels [1]. The acidic nature of tumors was later confirmed, and for many years, it was thought that tumors were characterized by both intracellular and extracellular acidic pH. More recently, measures based on magnetic resonance showed that cancer cells have a relatively normal, or even more alkaline intracellular pH, but their extracellular pH is acidic [2]. Currently, there is evidence that cancer cell pH is between 7.0 and 7.4, comparable to normal cells, whereas extracellular pH ranges between 6.9 and 7.0, although values lower than 6.0 have been reported [3]. Initially, extracellular acidification was considered the consequence of the high glycolysis rate and lactic acid production in tumor cells. Later, it was found that tumors that are not characterized by a high glycolytic rate also display an acidic extracellular environment [4]. These tumors have an elevated metabolic rate, with an elevated production of CO_2_. CO_2_ is converted within cells into H_2_CO_3_ by a class of enzymes known as carbonic anhydrases. H_2_CO_3_ in turn rapidly dissociates into H^+^ and $HCO_{3}^{-}$, contributing to acidification. Cells need to eliminate the intracellular excess of H^+^ and $HCO_{3}^{-}$, as well as lactic acid, to prevent intracellular acidification.

Low extracellular pH has been associated with several advantages for cancer cells. In vivo, acidic pH supports cancer cells invasiveness and promotes metastasis, at least in part by favoring extracellular matrix degradation and immune evasion [5,6]. On the other hand, low intracellular pH is dangerous for cell survival, and the maintenance of an adequate intracellular pH is fundamental for all cells, including cancer cells, to protect them from cell death [7]. Intracellular pH is regulated by several mechanisms. To effectively remove H^+^ from cells, CO_2_ must be preferentially hydrated in the extracellular space, because CO_2_ hydration into H_2_CO_3_ within cytosol would de facto maintain equivalent H^+^ levels. $HCO_{3}^{-}$ can be trapped extracellularly when CO_2_ hydration is catalyzed by carbonic anhydrases localized on the external leaflet of the plasma membrane, such as carbonic anhydrase IX, whose expression is considered a biomarker of extracellular pH acidification [8,9]. However, not all CO_2_ can be transported outside the membrane as a gas, due to poor solubility in H_2_O. Therefore, part of the CO_2_ is hydrated intracellularly, and then H^+^ and bicarbonate must be exported extracellularly by different transporters. Bicarbonate transporters are grouped into two solute carrier (SLC) families, SLC4 and SLC26. Proteins classified in both groups can be either Na^+^-independent or Na^+^-dependent, as they also include Na^+^-$HCO_{3}^{-}$ cotransporters [10,11]. H^+^ are extruded by monocarboxylate transporters (MCT) that extrude H^+^ or Na^+^ and monocarboxylic acids such as lactate and pyruvate outside the cell [12]. H^+^ are also eliminated by sodium/hydrogen exchangers (NHEs), a family of transporters using the sodium gradient across the plasma membrane to extrude protons produced by metabolism [13]. Voltage gated sodium channels (VGSCs) participate in the regulation of NHE-1 exchanger protein, increasing proton extrusion [14]. Finally, the cytosolic concentration of H^+^ can be decreased by sequestration within acidic organelles such as endosomes and lysosomes, thanks to the activity of vacuolar ATPases. These proton pumps are also localized on the plasma membrane, and their activity, together with lysosomal exocytosis, further contributes to extracellular acidification [15,16,17].

The evolution of a multiplicity of mechanisms extruding H^+^ and preventing an increase in intracellular acidity is an indication that intracellular acidification is toxic for cells, and cells need to export acid molecules to prevent it and avoid apoptosis [18]. For this reason, the reversal of cancer cells’ pH gradient, i.e., the promotion of intracellular acidity and extracellular alkalosis, has recently become a therapeutic strategy for cancer [19,20], and membrane transport inhibitors have entered clinical trials [21]. It has been proposed that cancer cell pH can be attacked from three different perspectives (triple edged approach) [22], i.e., by increasing intracellular lactic acid concentration via mitochondrial function inhibition (mitochondrial poison), by blocking proton extrusion acting on transporters, and by inducing the release of H^+^ from intracellular acidic organelles, such as lysosomes, via lysosomal membrane permeabilization (lysosomal poison).

Systems of molecules of vegetal origin and synthetic compounds have been screened for properties related to the regulation of cancer cell metabolism [23]. Here, we investigated systems of molecules of vegetal origin in prostate cancer cell models to evaluate their ability to promote the reversal of cancer cell pH toward a more accentuated intracellular acidification and extracellular alkalinization, as well as their properties in terms of modification of organelle pH. We provide evidence that systems of molecules of vegetal origin can affect cellular pH in vitro, providing a proof-of-principle that they can be used to promote pH normalization in cancer cells. In addition, they are also compatible with chemotherapeutic protocols currently being used.

## 2. Results

### 2.1. Analysis of Cell Viability

Systems of molecules of vegetal origin, prepared as described in Section 4, are reported in Table 1.

**Figure 1:**
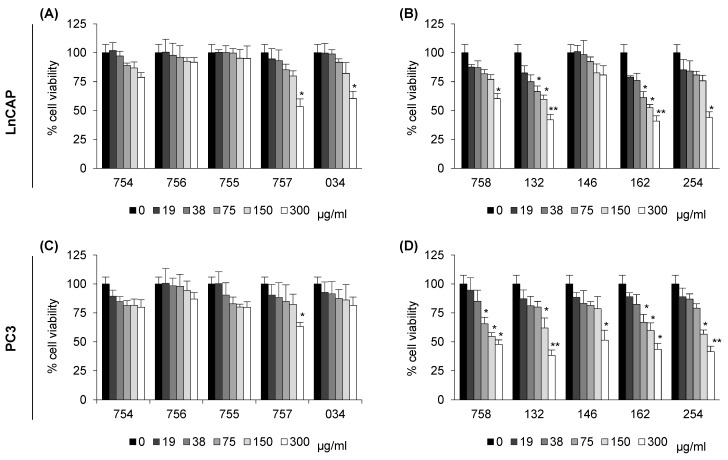
**Viability of LnCAP (A,B) and PC3 (C,D) cells upon exposure to systems of molecules of vegetal origin.** Cells were treated for 24 h with systems of molecules of vegetal origin, then resuspended either in cell culture medium panel (**A**,**C**) or in DMSO panel (**B**,**D**), at the indicated concentrations. Cell viability was determined by MTT assay. Data is expressed as a percentage of cell survival, with respect to vehicle-treated cells as control (set 100). They are mean ± SE of three independent experiments, each performed in triplicate. * *p* < 0.05, ** *p* < 0.01 were calculated by *t*-test. Systems of molecules of vegetal origin prepared as summarized in Table 1 were tested: long pepper (254), nutmeg (132), feverfew (162), filipendula (757), laurel freeze (034), artichoke (758), turmeric (755), and poplar freeze (146). Sodium citrate (754) and sodium bicarbonate (756) were used as controls.

They were resuspended in cell culture medium (755, 757, 034) and sodium citrate (754) and sodium bicarbonate (756) as controls, or in DMSO (758, 132, 146, 162, 254), then diluted at the indicated concentration and administered to Lymph Node Carcinoma of the Prostate cells (LnCAP, upper panels) and Prostate Cancer cells (PC3, lower panels). Those resuspended in cell culture medium are reported in Figure 1A,C, and those in DMSO in Figure 1B,D. After 24 h incubation, MTT solution was added to evaluate cell viability. In Figure 1, all concentrations tested are reported. Interestingly, at 38 µg/mL, no systems of molecules induced a cell viability significantly lower than control.

### 2.2. Measure of Intracellular pH

**Figure 2:**
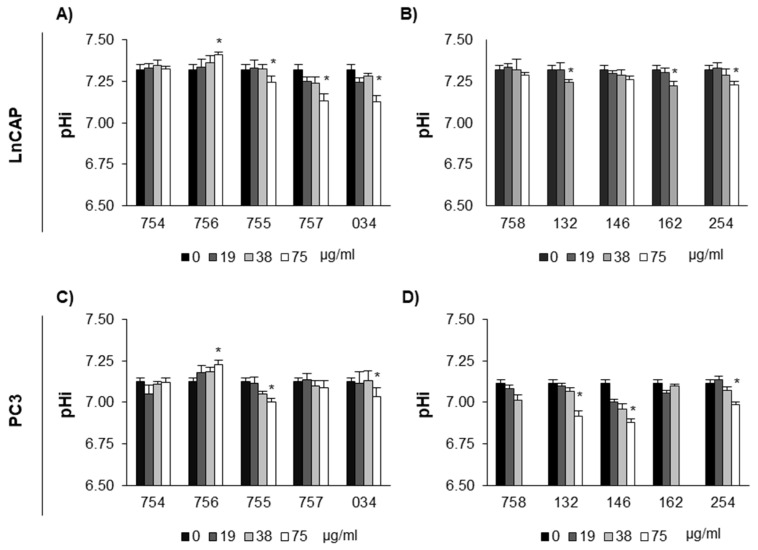
**Effect of systems of molecules of vegetal origin on intracellular pH (pHi) in LnCAP (A,B) and PC3 (C,D) prostate cancer cells**. Intracellular pH was measured with BCECF-AM probe. Cells were loaded with 2 µM BCECF-AM and their fluorescence read (ex 440 nm and 490 nm, em 535 nm). Cells were then incubated with plant extracts, resuspended either in cell culture medium as in panel (**A**,**C**) or in DMSO, as in panel (**B**,**D**) for 1 h at the indicated concentrations and fluorescence read (ex 440 nm and 490 nm, em 535 nm). The pHi was calculated based on an in situ calibration curve. Each value represents the mean ± SE of three determinations, each performed in triplicate. * *p* < 0.05 with respect to vehicle-treated cells was considered statistically significant by *t*-test. Systems of molecules of vegetal origin prepared as summarized in Table 1 were tested: long pepper (254), nutmeg (132), feverfew (162), filipendula (757), laurel freeze (034), artichoke (758), turmeric (755), and poplar freeze (146). Sodium citrate (754) and sodium bicarbonate (756) were used as controls.

LnCAP and PC3 cells were treated with systems of molecules at concentrations not affecting cell viability based on MTT assay to examine changes in intracellular pH (pHi). Results showed (Figure 2) that several tested plant extracts have a dose-dependent acidifying effect, but not 754 (sodium citrate) and 756 (sodium bicarbonate). Indeed, 756 was the only compound tested with a clear and dose-dependent alkalinizing effect, whereas 754 has no effect at all, indicating that the combined effect of Na^+^ and bicarbonate favors intracellular alkalinization in our experimental conditions [24]. In the case of systems of molecules, for both LnCAP and PC3 cell lines 755, 034, and 254 showed significant acidifying effect.

### 2.3. Measure of Extracellular pH

**Figure 3:**
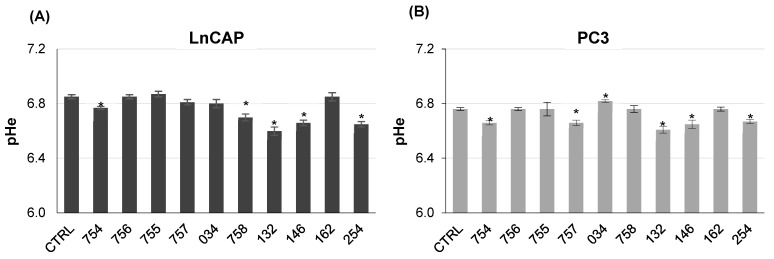
**Effect of systems of molecules of vegetal origin on extracellular pH (pHe) in LnCAP (A) and PC3 prostate cancer cells (B).** Cells were treated for 24 h with plant extracts at 38 µg/mL concentration in cell culture medium w/o a buffering system. Medium was then collected and centrifuged to eliminate cells and cell debris. Extracellular pH was measured with a pH meter. Values represent the average of six determinations, each one in quadruplicate. Bars represent systems of molecules diluted in cell culture medium and incubated for 24 h at 37 °C in humidified atmosphere. Each value represents the mean ± SE of six determinations, each one in quadruplicate. * *p* < 0.05 with respect to vehicle-treated cells as control.

LnCAP and PC3 cells were treated for 24 h with 38 µg/mL concentration of the plant extracts, diluted in a cell medium devoid of buffering system, therefore allowing cells to change their extracellular pH. The concentration was chosen based on MTT assay, because it allowed us to maintain a cell viability above 80% for 24 h, with no significant differences as compared to the control. Figure 3 reports the acidifying effect of systems of molecules incubated with the cell culture medium for 24 h at 37 °C in humidified atmosphere in the presence of cells (Figure 3), with respect to untreated cells as control. When compared to untreated cells, a few plant extracts (132, 146, 254) showed acidifying effects towards both cell lines, whereas 757 showed acidifying properties only in PC3 and 758 only in LnCAP cells, thus providing evidence of a cell-specific response towards specific systems. On the other hand, alkalinization was detected in the cell culture medium of PC3 cells treated with 034.

### 2.4. Measure of Organelle pH

**Figure 4:**
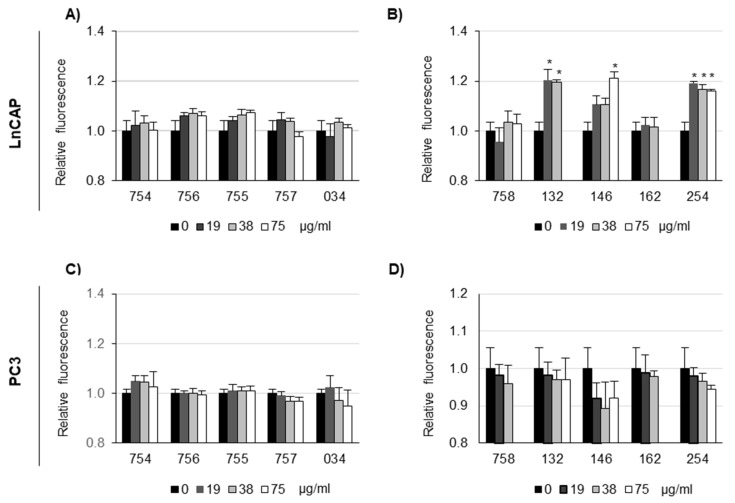
**Effect of systems of molecules of vegetal origin on lysosomal organelle pH in LnCAP (A,B) and PC3 (C,D) prostate cancer cells.** Organelle pH was measured using LysoSensor DND-189. Cells were preloaded with 1 µM probe diluted in HBSS + 20 mM HEPES, and fluorescence was measured (ex 443, em 505); then, cells were treated for 90 min with systems of molecules, resuspended either in cell culture medium as in panel (**A**,**C**) or in DMSO, as in panel (**B**,**D**), then diluted in HBSS + 20 mM HEPES, at the indicated concentrations, and fluorescence was measured (ex 443, em 505). The ratio between the fluorescence at time 0 and the fluorescence after 90 min was calculated, and the relative level of fluorescence with respect to vehicle-treated cells, set as 1, is reported. Each value represents the mean ± SE of four determinations, each one in triplicate. * *p* < 0.05 with respect to vehicle-treated cells was calculated by *t*-test. Systems of molecules of vegetal origin prepared as summarized in Table 1 were tested: long pepper (254), nutmeg (132), feverfew (162), filipendula (757), laurel freeze (034), artichoke (758), turmeric (755), and poplar freeze (146). Sodium citrate (754) and sodium bicarbonate (756) were used as controls.

LnCAP and PC3 cells were treated with systems of molecules at increasing concentrations to evaluate organelle pH. LysoSensor Green DND-189 was used to qualitatively evaluate pH changes in acidic organelles of the endosomal/lysosomal system, as this probe becomes more fluorescent in acidic environments and less fluorescent in alkaline environments [25]. Cells were preloaded with the probe diluted, and fluorescence was measured (F0); then, they were treated for 90 min with systems of molecules, at the indicated concentrations, and fluorescence was measured again (F90). The ratio F0/F90 was calculated, and it was expected to decrease in case of alkalinization (F0 > F90, so F0/F90 > 1) and to increase in case of acidification (F0 < F90, so F0/F90 > 1). The relative level of fluorescence with respect to untreated cells as control, set 1, was reported. Results (Figure 4) showed a cell-specific effect of systems of molecules. In fact, no significant alkalinization effect could be detected for PC3 cells, whereas for LnCAP cells, alkalinization could be detected for 132, 146 and 254 samples.

## 3. Discussion

Intracellular alkalosis and extracellular acidosis are two pathological features associated with malignant cells. They offer advantages in terms of invasiveness and proliferation, although additional mechanisms cannot be excluded [26,27]. Organelle pH also represents an important feature of cancer cells, as lysosomal-mediated cell death in neoplastic cells is considered a promising strategy to overcome chemoresistance, especially in cells able to bypass apoptotic cell death [28,29]. Different classes of natural molecular complexes are receiving a lot of attention for their properties, in terms of their ability to modulate cancer metabolism and cancer-associated processes, such as angiogenesis: the main ones are polyphenols and flavonoids [30]. Systems of molecules of vegetal origin represent an important group of bioactive compounds derived from plant-based foods and beverages with known biological activity in cells. They have already shown efficacy in the treatment of in vivo models of inflammation [31]. In this study, we have investigated the role of systems of molecules of vegetal origin to modulate cancer cell intracellular and extracellular pH, as well as lysosomal pH, using in vitro prostate cancer cells as models.

Cancer cells are characterized by the so-called pH inversion, i.e., a higher intracellular pH and a lower extracellular pH [32,33], so we looked for systems of molecules of vegetal origin able to decrease intracellular pH and increase extracellular pH. Our results provide evidence that a few of these systems (034, 755, 132, 254) could promote the acidification of intracellular pH in both PC3 and LnCAP prostate cancer cell models at concentrations that do not significantly affect cell viability. For other systems, the effect was cell-type specific, i.e., 146 was shown to promote intracellular acidification only in PC3 and 162 only in LnCAP. As for extracellular pH, only one system of natural origin (034) was able to induce extracellular alkalinization, and in PC3 cell type, whereas no effect on LnCAP was observed. This finding shows that several systems were able to prompt intracellular acidification contrasting the intracellular alkalinization associated with malignant behavior, but it is more difficult to obtain positive hits screening for systems of natural origin inducing extracellular alkalinization. In addition, our data show that it is a demanding task to find a single system of natural origin able to induce both intracellular acidification and extracellular alkalinization in both cell types, as only 034 was able to promote intracellular acidification in both cell types and extracellular alkalinization, but only in PC3 with no effect on LnCAP. In addition, it must be considered that concentrations not significantly affecting cell viability were estimated only by cell viability assay, and further estimation of live/dead cells ratio could also offer additional information.

We then tested the capacity of systems of molecules of vegetal origin to modulate lysosomal pH. Our data provides evidence that a few systems of natural origin can promote lysosomal alkalinization, i.e., 132, 146 and 254, but only on LnCAP cells, whereas no system was effective in PC3 cells. Androgen-dependent LNCaP cells are well differentiated and less invasive as compared to PC3, which are androgen-independent, less differentiated, and more invasive [34,35]. Their lysosomal pH clearly shows different sensitivity to systems of molecules of vegetal origin, but the molecular reason underlying these differences is unclear and should be further investigated.

Due to the relevant role that pH inversion plays in tumor biology in terms of proliferation, survival, invasion and metastasis, it is not surprising that targeting the pH inversion is a very active area of therapeutic research. Our results show that selected systems of natural origin show important properties in terms of modulation of intracellular, extracellular and organelle pH. However, our study has limitations that need to be addressed by further research. First, our approach using plate assay is potentially scalable, but it is limited to in vitro assays on cultured cells, so the development of additional assays and the in vivo testing are required to confirm these results. Second, it is difficult to obtain all the convenient properties and a wide spectrum effect with only one system of natural origin. For this reason, the screening of additional systems of natural origin, as well as of mixture of systems of natural origin, could be analyzed in future studies to reach more suitable formulations. Third, it is also worth considering that our approach did not investigate single active principles included in systems of molecules of vegetal origin, because our aim was to test the effect of the whole systems of molecules of vegetal origin. Understanding the molecular mechanisms and the pharmacological targets behind the effect of systems of molecules with a reductionist approach, trying to unravel individual active molecules within these systems, could represent an additional objective, but it is worth remembering that biological activity could be possibly due to synergistic phenomena. Our study provides evidence that these systems of molecules of vegetal origin, as a whole, can be considered, and further developed as, interesting agents in co-adjuvating anti-cancer therapies.

## 4. Materials and Methods

### 4.1. Materials

Ethanol food grade (96.4%, obtained from wheat) and purified water (obtained by means of an industrial plant for water treatment) were the solvent used for the extraction processes. Sodium citrate (ABO-AR-2016-754) was a food grade dihydrate salt and was purchased from Faravelli (via Medardo Rosso, 8—Milano, Italy). Sodium bicarbonate (ABO-AR-2016-756) was a food grade salt (purity 99–101%) from nahcolite and was purchased from Natural Soda (3200 County Road 31, Rifle, CO, USA).

### 4.2. Freeze-Dried Herbal Extracts

The extracts used, their codes, and details on the extraction procedure are listed in Table 1. All freeze-dried herbal extracts were produced by Aboca Spa (Sansepolcro, AR, Italy) according to the following extraction procedures.

*Procedure 1_ applied to Long pepper, Nutmeg, Feverfew.* The dried, ground plant materials of each species were introduced in the extractor together with the solvent and extracted, protected from the light, by mechanical stirring at constant temperature. Depending on the characteristics of the herbs, the extraction was performed with different water–ethanol percentage and herb–solvent ratios. After 6–7 h, the plant hydro-alcoholic extracts were filtered to remove the depleted herb and concentrated under vacuum to evaporate ethanol.

*Procedure 2_ applied to Laurel.* The dried, ground plant materials were introduced in the Naviglio extractor and extracted by the solvent at constant temperature, according to a standard procedure [36]. After 6 h, the plant hydro-alcoholic filtered extract was collected and concentrated under vacuum to evaporate ethanol.

*Procedure 3_ applied to Artichoke, Turmeric.* The dried, ground plant materials of each species were introduced in the perforated basket present inside the extractor. The solvent was added and extraction proceeds maintaining the solvent recirculating by means of a pump, protected from the light. During the extraction the solvent is continuously pumped from the bottom of the extractor and dropped from above onto the plant material. Depending on the characteristics of the herbs, the extraction was performed with different water–ethanol and herb–solvent ratios. After 8 h, the plant hydro-alcoholic extracts were filtered to remove the depleted herb and concentrated under vacuum to evaporate ethanol.

*Procedure 4_ applied to Poplar.* The extract was prepared according to patent n° WO2017203414. The dried, ground plant materials were introduced in the perforated basket present inside the extractor. The solvent was added and extraction proceeds maintaining the solvent recirculating by means of a pump, protected from the light. During the extraction the solvent is continuously pumped from the bottom of the extractor and dropped from above onto the plant material. The extraction was performed at first with ethanol 85%. After 6 h, the plant hydro-alcoholic extract was filtered to remove the herb first extracted. The herb so treated was extracted again with ethanol 13%. After 6 h, the plant hydro-alcoholic extract was filtered to remove the depleted herb. The two hydro alcoholic extracts were mixed, the precipitated was eliminated and the supernatant was concentrated under vacuum to evaporate ethanol.

All the aqueous concentrates obtained after ethanol evaporation were rapidly freeze-dried, under vacuum, without the use of excipients, at temperatures lower than −50 °C. Water was removed by sublimation and the resulting freeze-dried extracts were stored at room temperature protected from moisture and light until use.

### 4.3. Cell Culture and Treatments

PC3 and LnCAP cells were maintained in RPMI supplemented with 10% fetal bovine serum (FBS), 50 U/mL penicillin, and 50 µg/mL streptomycin. Cells were cultured at 37 °C in a humidified atmosphere with 5% CO_2_ and passaged using 0.5% trypsin-EDTA. Systems of molecules were resuspended either in cell culture medium (757, 755, 034) or in DMSO (758, 132, 146, 162, 254), at the concentration of 30 mg/mL, except for 757 (10 mg/mL). At these concentrations, no pellet was observed after centrifugation at 10,000× *g* for 15 min at RT. Two salts (sodium citrate as 754 and sodium bicarbonate as 756) were used as control. Resuspended extracts were vortexed for 2 min at RT, sonicated for 5 min with a Saniprep 150 sonicator (MSE, London, UK), then centrifuged at 5000 rpm for 10 min at RT. Supernatants were transferred in a new tube, stored at −20 °C and used for cell treatments.

### 4.4. MTT Assay

For this assay, 2 × 10^4^ cells were seeded in a 96-well multiplate 24 h before their treatment with systems of molecules of vegetal origin diluted in complete cell culture medium. After 24 h incubation with systems of molecules of vegetal origin, MTT solution was added to cells at the final concentration of 0.2 mg/mL for 3 h at 37 °C. The solution was eliminated, the dark blue formazan crystals were solubilized with DMSO o/n at RT, and then the absorbance was read at 570 nm using an Infinite 200 Pro microplate reader (Tecan, Zurich, Switzerland).

### 4.5. Measurement of Intracellular pH: BCECF-AM Assay

For this assay, 3 × 10^4^ cells were plated in a 96-well black plate with a clear bottom. After 24 h, the medium was removed, and cells washed 3 times with HBSS. BCECF-AM (Life Technologies, Carlsbad, CA, USA) was resuspended at 1 mM concentration in rigorously anhydrous DMSO. The BCECF-AM was diluted at 2 µM concentration in HBSS and added to cells that were incubated for 15 min in the dark. Cells were washed with HBSS, and their fluorescence measured at excitation wavelength of 440 nm (isosbestic point, pH independent) and 490 nm. In both cases, the emission wavelength was 535 nm. Then, cells were treated with systems of molecules diluted in HBSS + 20 mM HEPES. Systems were incubated at 37 °C w/o CO_2_ up to 1 h 30 min, and fluorescence was measured as indicated above (ex 440 and 490 nm, em 535 nm); then, 1 h incubation was chosen as the best option in terms of signal-to-noise ratio. For each assay, an in situ calibration curve was carried out. Briefly, calibration solutions at different pH and containing 27 µM nigericin were prepared (135 mM KCl, 2 mM K_2_HPO_4_, 20 mM HEPES, 1.2 mM CaCl_2_, 0.8 MgSO_4_, adjusted at pH 5.5, 6.0, 6.5, 6.75, 7.0, 7.25, 7.5, 8.0, 8.5 with HCl or KOH) and added to cells. These were incubated in the dark for 15 min at 37 °C, and then fluorescence was measured (ex 440 and 490 nm, em 535 nm). Intracellular pH (pHi) was then calculated following the equation reported by Graber [35] and considering that the pKa of BCECF is 7.0 [37].

### 4.6. Measurement of Extracellular pH

For this assay, 2 × 10^5^ cells were plated in a 6-well multiplate. After 24 h, the culturing medium was removed, and cells washed with PBS. Systems of molecules were diluted in complete RPMI medium adjusted at pH 7.2 with HCl but devoid of a further buffering system. Cells were incubated for 24 h at 37 °C in a humidified atmosphere. Medium was then collected, centrifugated at 1000× *g* per 5 min to eliminate cell debris and pH was measured with a pH-meter (Mettler-Toledo, Columbus, OH, USA).

### 4.7. Measurement of Organelle pH

LysoSensor Green DND-189 was used to qualitatively evaluate pH changes of acidic organelles of the endosomal/lysosomal systems, as this probe becomes more fluorescent in acidic environments and less fluorescent in alkaline environments [38,39]. Cells were plated in a 96-well clear-bottom black plate. After 24 h, culturing medium was removed, and fresh complete medium containing 1 µM LysoSensor DND-189 (Life Technologies) was added and incubated at 37 °C in the dark. Excess probe was washed with HBSS, and fluorescence was measured (ex 443, em 505) in a microplate reader Infinite 200 Pro microplate reader (Tecan, Zurich, Switzerland). Then, cells were treated with systems of molecules diluted in HBSS + 20 mM HEPES for up to 120 min, and fluorescence was measured (ex 443, em 505); then, 90 min incubation was chosen as the best option in terms of signal-to-noise ratio. The relative level of fluorescence (vehicle-treated cells versus treated cells) was calculated. Systems of molecules diluted in HBSS + 20 mM HEPES fluorescence were also measured and did not display significant values as compared to HBSS + 20 mM HEPES alone.

### 4.8. Statistical Analysis

Quantitative data is presented as mean  ±  SE of at least three independent experiments. Statistical comparison was performed using Student’s *t*-test. Differences were considered statistically significant when *p* < 0.05.

## Figures and Tables

**Table 1:** **Description of the systems of molecules of vegetal origin.** The code, binomial name, plant part, extraction solvent, plant-to-solvent ratio, extraction time and temperature, and freeze-dried extract yield are reported. The last 3 numbers of the code were used to indicate the systems of molecules of vegetal origin in text and figures.

Description	Code	Binomial Name	Plant Part	Extraction Solvent	Plant-to-Solvent Ratio	Extraction Time	Extraction Temperature	Freeze-Dried Extract Yield
**Long pepper**	2016-**254**	*Piper longum* L.	Grains	Ethanol 70%	1/2	7 h	40 °C	7%
**Nutmeg**	2016-**132**	*Myristica fragrans* Houtt	Seed	Ethanol 70%	1/5	7 h	40 °C	4%
**Feverfew**	2016-**162**	*Tanacetum parthenium* L.	Flowers	Ethanol 70%	1/8	7 h	40 °C	16%
**Filipendula**	2016-**757**	*Filupendula vulgaris* Hill	Flowered aerial parts	Ethanol 50%	1/10	6 h	40 °C	18%
**Laurel freeze**	2014-**034**	*Laurus Laurus nobilis* L.	Leaf	Ethanol 70%	1/6	7 h	40 °C	15%
**Artichoke**	2016-**758**	*Cynara scolymus* L.	Leaf	Ethanol 50%	1/10	8 h	50 °C	17%
**Turmeric**	2016-**755**	*Curcuma longa* L.	Root	Ethanol 40%	1/8	8 h	50 °C	7%
**Poplar freeze**	2013-**146**	*Populus nigra* L.	Buds	1-Ethanol 85% 2-Ethanol 8%	1–1/6 2–1/6	1–6 h 2–6 h	50 °C	12%

## Data Availability

Data is contained within the article.
